# Modeling of H_2_ Permeation through Electroless Pore-Plated Composite Pd Membranes Using Computational Fluid Dynamics

**DOI:** 10.3390/membranes11020123

**Published:** 2021-02-09

**Authors:** Alberto Fernández, Cintia Casado, David Alique, José Antonio Calles, Javier Marugán

**Affiliations:** 1 Department of Chemical and Environmental Technology, Rey Juan Carlos University, C/Tulipán s/n, 28933 Móstoles, Spain; alberto.fernandezf@urjc.es (A.F.); cintia.casado@urjc.es (C.C.); 2 Department of Chemical, Energy and Mechanical Technology, Rey Juan Carlos University, C/Tulipán s/n, 28933 Móstoles, Spain; david.alique@urjc.es (D.A.); joseantonio.calles@urjc.es (J.A.C.)

**Keywords:** composite membrane, palladium, electroless plating, hydrogen, gas separation, source–sink, Darcy–Forcheimer, permeation rate, multiphysics modeling, experimental validation

## Abstract

This work focused on the computational fluid dynamics (CFD) modeling of H_2_/N_2_ separation in a membrane permeator module containing a supported dense Pd-based membrane that was prepared using electroless pore-plating (ELP-PP). An easy-to-implement model was developed based on a source–sink pair formulation of the species transport and continuity equations. The model also included the Darcy–Forcheimer formulation for modeling the porous stainless steel (PSS) membrane support and Sieverts’ law for computing the H_2_ permeation flow through the dense palladium film. Two different reactor configurations were studied, which involved varying the hydrogen flow permeation direction (in–out or out–in). A wide range of experimental data was simulated by considering the impact of the operating conditions on the H_2_ separation, such as the feed pressure and the H_2_ concentration in the inlet stream. Simulations of the membrane permeator device showed an excellent agreement between the predicted and experimental data (measured as permeate and retentate flows and H_2_ separation). Molar fraction profiles inside the permeator device for both configurations showed that concentration polarization near the membrane surface was not a limit for the hydrogen permeation but could be useful information for membrane reactor design, as it showed the optimal length of the reactor.

## 1. Introduction

The use of hydrogen as an energy vector has a promising future due to its high energy density per mass unit and its viable production from a wide variety of feedstocks. It includes traditional hydrocarbons derived from fossil fuel and biomass [1], residual hydrocarbons or wastes [2], and even other molecules out of the carbon cycle, such as ammonia [3] or water [4]. Despite the increasing interest in developing new renewable production routes, most of the currently available hydrogen is obtained by traditional routes from fossil fuels, mainly via methane steam reforming [5] and the water–gas shift reaction [6]. In these processes, hydrogen is usually mixed with other gases, such as CO, CO_2_, N_2_, or steam, among others, and further downstream, separation steps are required to adjust its purity up to a certain level, depending on the final application [7]. At this point, it should be taken into account that these final purification steps can represent up to 50% of the total energy consumption for hydrogen production, thus representing a significant contribution to its final cost [8]. In recent years, the use of membrane separators has been proposed as an efficient alternative to traditional pressure swing adsorption systems due to their high efficiency, simple operation and maintenance, relatively low energy requirements, and scalability for both large and decentralized production [9]. Particularly, the use of dense Pd-based membranes has been widely studied due to its theoretically infinite H_2_ selectivity and relatively high permeability in comparison with other potential membrane materials [10]. Moreover, their excellent mechanical and thermal properties make possible their use as independent separators [9], which can be combined with the reaction step in a unique device called a membrane reactor [11]. For both cases, significant effort has been focused on reducing the palladium thickness [10] due to its high cost and the inverse relationship with permeate fluxes according to Sieverts’ law, which is widely used to describe the solution diffusion mechanism that governs the H_2_ permeation. In this context, supported membranes allow for achieving this target while maintaining enough mechanical strength [12]. Most researchers usually consider using ceramic and metallic porous substrates with particular characteristics, although an ideal solution has still not been reached. The ceramic ones provide excellent quality in terms of a smooth surface and narrow pore size distribution up to the nanometer scale at a reasonable cost. However, they present relative fragility when being handled, making their assembly difficult in conventional industrial devices, which are commonly made of stainless steel [13]. In contrast, the metallic ones, especially those made of stainless steel, guarantee a good sealing capability at the expense of the surface quality (high roughness and large pores) [14]. To mitigate these problems, a ceramic intermediate layer is frequently incorporated onto the external surface of porous metallic supports before depositing the Pd-based H_2_-selective film [15]. The technique used to incorporate this last film deserves to be briefly addressed since it usually determines the final cost and performance of the composite membrane. Among other alternatives, electroless plating (ELP), which is based on chemical redox reactions, can generate homogeneous and relatively thin Pd layers on both conducting and non-conducting substrates with a wide variety of geometries at low cost due to its experimental simplicity [16,17]. Diverse alternatives based on this technique have been proposed in the literature during recent years, including the electroless pore-plated (ELP-PP) technique, in which further mechanical resistance is reached. It is produced via the partial penetration of the palladium inside some of the support’s pores after feeding both the Pd source and reducing solutions into opposite sides of the porous substrate, as is widely described elsewhere [18]. However, some particular secondary effects of this partial penetration of the palladium inside the pores of the substrate have also been observed, with a slight deviation from the adjustment to the origin (0,0) proposed by the theoretical Sieverts’ law while depicting permeate fluxes against the pressure driving force raised to the power of 0.5 [18].

Computer-assisted simulations enable the parametric evaluation of the effects mentioned above, among others, as well as scaling up these new technologies at minimal cost, minimizing the investments in experimental tests. It also provides the possibility for optimizing the designs, being an ideal tool to enhance the results of non-mature research with potential industrial applications [19]. Specifically, these tools can improve the understanding of mass transfer in membrane module devices [20]. Some computational approaches to modeling H_2_ permeation through similar systems have previously been reported in the literature. The source–sink formulation has been commonly used to simulate H_2_ permeation, as Pd membranes constitute a discontinuity in the gas flux, which cannot be solved using the Navier–Stokes equations [21]. This formulation was used by Coroneo et al. [22,23]. They studied H_2_ permeation in a Pd-Ag alloy supported by a ceramic tube using Ansys Fluent by implementing H_2_ permeation flux due to the different mass transfer resistances offered by the support and the membrane. Chen et al. [24] applied a source–sink pair formulation to study the influence of the flow patterns of feed gas and sweep gas in the H_2_-permeation, produced by Water-Gas-Shift (WGS) reactions, through a dense Pd membrane using COMSOL (Comsol Inc., Burlington, MA, USA). Other computational fluid dynamics (CFD) studies of Pd-based membranes have been carried out recently. Ghasemzadeh et al. [25] analyzed the influence of temperature and pressure of the methanol steam reforming reaction that is used to produce hydrogen. The model was also used to compare silica and unsupported Pd-based membrane behaviors for hydrogen purification and to validate predictions with experimental data from the literature. Farangis et al. [21] developed some 2D models to study the influence of different flow patterns and Pd membrane geometries on the separation of hydrogen. They found that variations on the cross-section of the membrane can decrease the concentration polarization effect. Ben-Mansour et al. [26] simulated the implementation of a sweep gas to improve the H_2_-permeate flux through a Pd-based membrane for different binary mixtures. The same sink–source formulation has been used to model other types of membranes and feed gas mixtures. For example, Fatemi et al. [27] studied carbon dioxide permeation through a tubular zeolite membrane using the Ansys Fluent and Gambit platforms.

This work focused for the first time on the multiphysics modeling of a versatile permeator device based on electroless pore-plated composite Pd-membranes supported by tubular porous stainless steel (PSS) and the analysis of the concentration polarization on the membrane surface. Inner and outer regions of the annular separator could be configured as retentate or permeate sides (denoted as in–out or out–in configurations, respectively) for both the experimental and modeling approaches. A sandwich-type composite membrane, in which a Pd thin film was placed onto the external surface of a sintered 316L PSS support, was located between these two regions. The support was modeled as a porous zone. A source–sink pair formulation was implemented for the Pd film for computing the hydrogen separation under various operating conditions (feed pressure and H_2_ content in the feed stream). The model was validated by comparing all results with the ones obtained in an experimental campaign.

## 2. Materials and Methods

### 2.1. Pd Membranes: Preparation and Basic Characterization

All composite Pd membranes collected in this study were prepared on tubular PSS supports with symmetric structure, porosity of ≈20%, and 0.1 μm media grade, and were provided by Mott Metallurgical Corp. (Farmington, CT, USA). These raw supports had an external diameter of 0.5 inches and a wall thickness of around 1.9 mm. The original tubes were cut into small pieces 30 mm in length, cleaned, and calcined in air at 873 K for 12 h to generate a first intermediate layer that was formed by Fe-Cr oxides, as described in previous studies [28]. An additional CeO_2_-based intermediate layer was then incorporated using vacuum-assisted dip-coating to reduce both the average pore mouth size and external roughness of the support by a further grade [29]. Finally, ELP-PP of the palladium was used to generate the H_2_-selective film, as reported in previous publications [30,31].

The basic morphology of these composite Pd membranes was analyzed via SEM analysis, including both surface and cross-sectional views. Moreover, a series of gas permeation experiments were carried out to determine both the permeability and H_2_ selectivity of the prepared membranes in a homemade device that was described in our previous publications [28,29]. A 316L SS cell rounded using an external electrical furnace contained the Pd membrane in the middle of two graphite O-rings to ensure an adequate sealing between both the retentate and permeate sides. The experimental procedure consisted of first heating in an inert atmosphere from room temperature to 723 K. The membrane’s permeation capacity was evaluated under a controlled pressure ranging from 1.25 to 3 bar when feeding N_2_ and H_2_, first as single gases and later in binary mixtures that ranged from 0.5 to 1 H_2_ molar fractions. It should be noted that the permeate could be collected either from the lumen or from the annular side of the system (out–in or in–out configurations, respectively). The permeate side was always maintained at atmospheric pressure throughout the entire set of experiments, independently of this configuration. In any case, the permeate was collected and measured with a drum-type gas flow meter with a minimum detection limit of 1 mL/h. All these results were used to validate the modeling results of simulations included in the present study.

### 2.2. Computational Model

#### 2.2.1. Geometry and Mesh of the Membrane Cell

**Figure 1:**
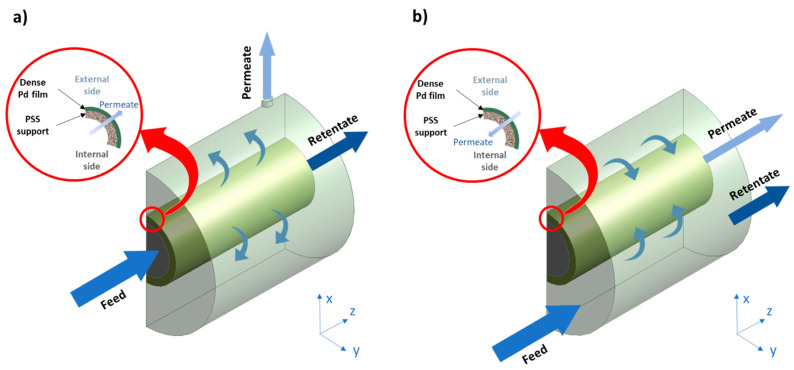
Schematic representation of the membrane permeator working in two different configurations: (**a**) in–out and (**b**) out–in. PSS: porous stainless steel.

The membrane permeator cell was modeled using CFD by considering its real dimensions: internal diameter of 30 mm, 30 mm in length, and a total volume of 21,200 mm^3^. As the geometry presented a symmetry plane, only half of the device was considered in the model. The tubular composite membrane had inner and outer diameters of around 11 mm and 13 mm, respectively, dividing the model geometry into three different regions, as shown in Figure 1. Depending on the flow direction through the membrane module, two different configurations were modeled. In the in–out configuration (Figure 1a), the gas was fed into the reactor through the inner region of the tubular membrane, collecting the retentate stream from the opposite side along the z-direction and the permeate stream in the annular zone. Thus, the H_2_ first reached the porous media (modified PSS with intermediate layers) and then the fully dense Pd film. A contrary permeation scheme was considered for the out–in configuration (Figure 1b), where the feed stream was introduced in the cell through the outer region. Therefore, the fed H_2_ was in direct contact with the outer surface of the Pd film and the permeate had to go through this layer and then the porous media before reaching the lumen bulk gas phase.

**Figure 2:**
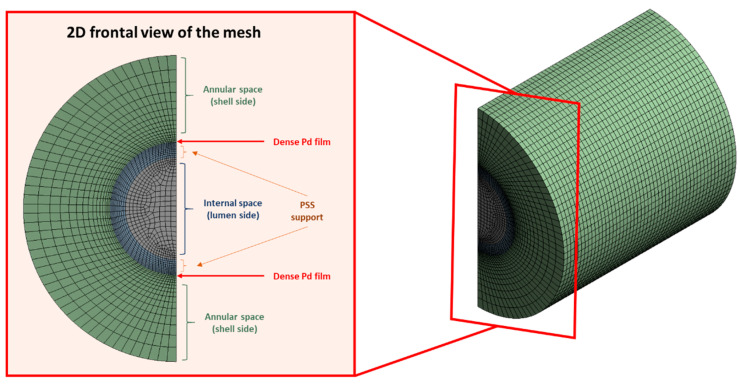
Illustrative mesh details for the membrane permeator for the out–in configuration.

A conformal mesh was established for the geometry interfaces to reduce the computational cost. The mesh was carefully refined close to the PSS and dense palladium region. This mesh slightly varied for the two different configurations considered in the present study, with 74,935 and 78,694 elements for the in–out and out–in alternatives, respectively. This number of cells was high enough to give mesh-independent results. An example of the established mesh for the out–in configuration is presented in Figure 2, with this being analogous to the other configuration, namely, in–out.

#### 2.2.2. Porous Support Modeling

The porous zone, corresponding to the PSS support, was implemented using the Darcy–Forcheimer model. This formulation provides accurate predictions for relatively low-pressure drops by adding a sink term (*S_i_*) to the fluid flow equations [32]. Its implementation in the CFD platform allows for calculating the variation of the velocity due to the effect of the membrane in each spatial direction:

(1) $$S_{i}=-\left(R_{V}{}_{i}\mu v_{i}\;+R_{I}{}_{i}\frac{1}{2}\rho |\vec{v|}v_{i}\;\right)$$

where $S_{i}$ is the sink term, v the velocity, μ the viscosity, ρ the density, and $R_{V}$ and $R_{I}$ are, respectively, the viscous and the inertial vectors of resistance coefficients (m^−1^) for the *i*th spatial direction.

Viscous and inertial resistance coefficients for each direction were calculated using:

(2) $$R_{V}=\frac{150{\left(1-\varepsilon \right)}^{2}}{\Phi^{2}D_{p}\varepsilon^{3}}$$

(3) $$R_{I}=\frac{2\times 1.75\left(1-\varepsilon \right)}{\Phi D_{p}\varepsilon^{2}}$$

where ε is the stainless steel’s porosity, $D_{p}$ is the particle diameter, and Φ is the particle sphericity.

**Figure 3:**
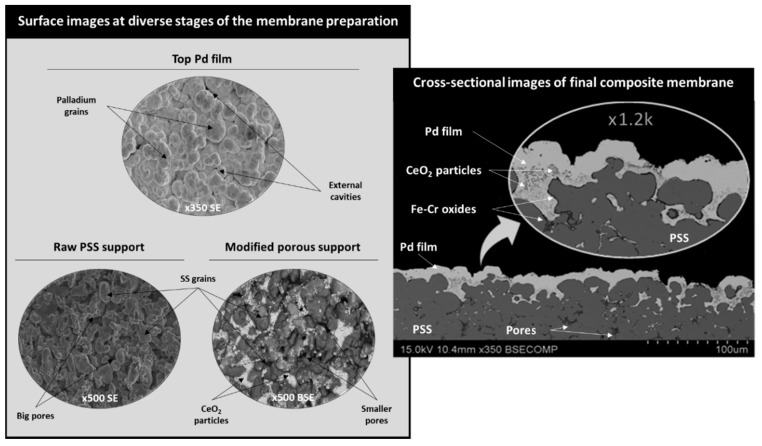
Morphology of the considered composite Pd-membrane for modeling: surface and cross-sectional SEM images at diverse stages of the membrane fabrication. BSE: back-scattering electrons, SE: secondary electrons, SS: stainless steel.

According to commercially available data, the porosity of commercial 0.1 μm PSS supports is around 20%. This value was confirmed using SEM image segmentation and analysis with Digital Micrograph^®^ (Gatan, Pleasanton, CA, USA), obtaining values of ε = 21.48% and *D_p_* = 21 µm, as previously reported elsewhere [30]. Finally, the PSS particles’ sphericity was set to Φ = 0.9. As shown in Figure 3, the membrane was constituted by the agglomeration and syntherization of large steel particles with variable sizes and morphologies. The average particle diameter and porosity were directly calculated from the segmentation of SEM images, while an average sphericity value of Φ = 0.9 was estimated by matching experimental data of nitrogen flow through the membrane to the model predictions.

With these parameters, viscous and inertial resistance coefficients of 2.61 × 10^13^ m^−1^ and 3.15 × 10^6^ m^−1^ were calculated, respectively, for each spatial direction. A factor of 10^3^ was applied in the axial direction as the permeate flux crossed the porous steel in the driving force direction, which was radial to the geometry. These parameters were validated using permeation experiments of PSS supports before incorporating the Pd film with pure nitrogen at two different feed pressure values.

#### 2.2.3. H_2_ Permeation through the Bulk Palladium

Assuming that the Pd film was placed on the outer surface of the porous support (as previously described in Section 2.1) and the feed stream was introduced by the lumen of the tubular support (in–out configuration), the complete H_2_ permeation mechanism involved the following fundamental steps [33]: (i) external mass transfer in the gas phase, (ii) transport through the porous media, (iii) hydrogen adsorption on the internal metal surface, (iv) hydrogen dissociation into atomic hydrogen, (v) diffusion through the bulk palladium, (vi) hydrogen recombination on the outer side of the palladium film, (vii) hydrogen desorption from the metal surface, and finally, (viii) external mass transfer in the gas phase of the permeate side. For the out–in configuration, in which the gas was fed to the annular region, similar steps could be distinguished, although in a different sequence.

The H_2_ solution and diffusion through the bulk palladium is typically the controlling step when the Pd thickness is thicker than a few micrometers [34]. In these conditions, the total permeate H_2_ flux $\left(J_{H_{2}}\right)$ can be described using the well-known Sieverts’ law [35], in which it is defined as a function of the membrane´s permeance K (mol·s^−1^·m^−2^·Pa^−0.5^) and the difference between the permeate $\left(P_{H_{2},per}\right)$ and retentate $\left(P_{H_{2},ret}\right)$ pressures to the exponent *n* = 0.5 acting as a driving force between both sides of the Pd film:

(4) $$J_{H_{2}}=K\left(P_{H_{2},ret}^{0.5}-P_{H_{2},per}^{0.5}\right)$$

In this study, the Pd film was modeled as a theoretically zero-thickness wall. The introduction of source and sink terms $\left(S_{i}\right)$ in the continuity and species transport equations simulated hydrogen’s disappearance from one side and its appearance on the palladium wall’s opposite side. Source term formulation was considered for each cell near this layer. Thus, Sieverts’ law could be reformulated to transform the surface flow into a local volumetric source–sink pair $\left(S_{i}\right)$, as follows:

(5) $$S_{i}=\pm \frac{A_{c}}{V_{C}}K\left({\left(Y_{H_{2},\;ret}P_{ret}\right)}^{0.5}-{\left(Y_{H_{2},per}P_{per}\right)}^{0.5}\right)$$

where $A_{c}$ and $V_{C}$ are the cell area and volume, respectively, and $Y_{i}$ is the species mole fraction. Each parameter included in Equation (5) was taken from its respective cell near the Pd film for each simulation iteration. As the hydrogen concentration in the permeation side was always maintained, in theory, equal to 1 (assuming perfect selectivity to hydrogen), Equation (5) could be rewritten as follows:

(6) $$S_{i}=\pm \frac{A_{c}}{V_{C}}K\left({\left(Y_{H_{2},\;ret}P_{ret}\right)}^{0.5}-P_{per}^{0.5}\right)$$

The user-defined functions (UDFs) for the source and sink terms can be found as Supplementary Information. The experimental K values obtained for each considered H_2_ feed molar fraction ranging from 1 to 0.5 were used to evaluate the model performance at diverse pressure driving forces. Despite being considered a zero-thickness wall in the model geometry (for the sake of simplicity), the thickness of the Pd membrane, and its impact on the H_2_ permeation, was implicitly considered by the inclusion of the specific *K* values in the calculations. The same approach was followed with the CeO_2_ intermediate layer, whose effect was directly included in the experimental K values.

Here, it should be noted that most of the Pd composite membranes prepared using ELP-PP exhibited a deviation from the ideal Sieverts’ law, meaning that it was necessary to consider a certain value for the y-intercept instead of directly fitting to the origin (0,0). This deviation does not yet have a clear physical interpretation, although an additional resistance to the H_2_ transport due to the partial penetration of the palladium in some of the PSS pores was proposed in previous studies [18].

The *y*-intercept and *K* values for each configuration used in the CFD model are presented in the result section.

#### 2.2.4. Fluid Dynamics

Fluid dynamics calculations have been carried out through the combination of the continuity equation (Equation (7)) and the classical Navier–Stokes equation (Equation (8)):

(7) $$\nabla \left(\rho \bar{v}\right)=0$$

(8) $$\nabla \cdot \left(\rho \bar{v}\bar{v}\right)=-\nabla P+\nabla \cdot \overset{=}{\tau }+\rho \vec{g}$$

where ρ, $\bar{v}$, *P*, $\overset{=}{\tau }$, and $\vec{g}$ are the fluid density, velocity vector, pressure, viscous stress tensor, and gravitational acceleration, respectively.

The standard *k*–*ϵ* model was used to model the turbulent flow. This two-equation turbulence model considered the turbulent length and time scale by solving two separate transport equations for the turbulent kinetic energy (*k*) and its dissipation rate (*ϵ*). This standard model assumes an isotropic scalar turbulent viscosity, treats the flow as completely turbulent, and neglects the molecular viscosity. Therefore, it is only valid for fully turbulent fluids. The energy transport, the dissipation rate, and the turbulent viscosity are described by Equations (9)–(11), respectively [36]:

(9) $$\rho \frac{\partial k}{\partial t}+\rho U_{i}\frac{\partial k}{\partial x_{i}}=\frac{\partial }{\partial x_{j}}\left[\left(\mu +\frac{\mu_{t}}{\sigma_{t}}\right)\frac{\partial k}{\partial x_{j}}\right]+\tau_{ij}\frac{\partial U_{i}}{\partial x_{j}}-\rho \epsilon$$

(10) $$\rho \frac{\partial \epsilon }{\partial t}+\rho U_{i}\frac{\partial \epsilon }{\partial x_{i}}=\frac{\partial }{\partial x_{j}}\left[\left(\mu +\frac{\mu_{t}}{\sigma_{\epsilon }}\right)\frac{\partial \epsilon }{\partial x_{j}}\right]+\rho C_{1}S_{\epsilon }-\rho C_{2}\frac{\epsilon^{2}}{k+\sqrt{\nu \epsilon }}$$

(11) $$\mu_{t}=\rho \frac{C_{\mu }k^{2}}{\epsilon }$$

In these expressions, $\sigma_{k}$ and $\sigma_{\epsilon }$ are the turbulent Prandtl numbers for *k* and *ϵ*, respectively; $C_{1}$ and $C_{2}$ are the model empiric constants that can be found elsewhere [36]; $S_{k}$ and $S_{\epsilon }$ are the terms for user-defined contributions.

Simulations with the turbulent model were compared with those using the laminar one. Complete correspondence between both models was observed except in a few cases, where very slight differences were found.

#### 2.2.5. Boundary Conditions and Physical Properties

Hydrodynamics calculations were carried out by considering a three-dimensional, steady-state, and turbulent flow after solving both the continuity and classical Navier–Stokes equations. The fluid was assumed to be Newtonian, compressible, and isothermal (648 K). The thermophysical properties of the mixture were determined using the Peng–Robinson equation of state. The hydrogen diffusion coefficient was calculated using a correlation with diffusion coefficients values taken from the Aspen Plus^®^ V11 software (Aspentech, Bedford, MA, USA) at similar operating conditions to the experiments (pressure and temperature) as follows:

$$D_{H_{2}}\;=1.236\times 10^{-25}P^{4}-1.222\times 10^{-19}P^{3}+4.673\times 10^{-14}P^{2}-8.613\times 10^{-9}P+7.631\times 10^{-4}$$

where $D_{H_{2}}$ is the hydrogen diffusion coefficient (m^2^/s) and *P* is the pressure (Pa).

The viscosity of the mixture at the experimental temperature was calculated as a function of the hydrogen molar fraction according to:

$$\mu \;=-2.708\times 10^{-5}Y^{4}+2.902\times 10^{-5}Y^{3}-1.741\times 10^{-5}Y^{2}-3.113\times 10^{-7}Y+3.098\times 10^{-5}$$

where μ is the dynamic viscosity (Pa·s) and *Y* is the hydrogen molar fraction.

The inlet feed was set with a direction normal to the boundary, where the values ranged from 1.25 × 10^−4^ g/s to 5.57 × 10^−3^ g/s as a function of the hydrogen concentration in the mixture. The retentate pressure was set at the experiment feed pressure in both configurations. On the other hand, the permeate was fixed at atmospheric conditions. A no-slip boundary condition was imposed at all model walls. The interface between the porous substrate and geometry domains was set as an interior region.

#### 2.2.6. Convergence Criteria and Solution Methods

The segregated steady-state solver was used to resolve the governing equations. A second-order upwind discretization scheme was used, except for pressure, for which the standard method was selected. The SIMPLE (Semi-Implicit Method for Pressure Linked Equations) algorithm was chosen for the pressure–velocity coupling. The numerical solution’s convergence was ensured by monitoring the scaled residuals below 10^−6^ for the continuity and momentum variables. Additionally, the variables of interest were monitored at different computational domain surfaces as an indicator of convergence (at least 50 iterations without changes). The complete model was solved using ANSYS Fluent software v.17.0 (Canonsburg, PA, USA).

## 3. Results and Discussion

### 3.1. Fundamental Membrane Characterization

The composite membrane used in this study, as previously indicated, was 30 mm in length and had a 1.27 mm outer diameter, with an estimated external surface of 1.14 × 10^3^ mm^2^. This value was calculated by assuming a perfect cylindrical geometry with the above-mentioned values of length and diameter. This membrane was prepared as diverse stacked layers containing a first PSS support modified with Fe-Cr oxides, an intermediate layer formed with mesoporous CeO_2_ particles, and a top Pd film to reach selectivity to hydrogen during its use. Despite being modeled as a zero-thickness wall, the real Pd film had an average thickness of 10.2 μm, which was estimated using gravimetric analyses by assuming a homogeneous deposition. Nevertheless, the observation of the membrane’s surface variation during its synthesis is important for understanding the structure of the modeled system correctly before going into depth with the mathematical approaches and obtained results. In this context, Figure 3 contains diverse surface micrographs taken at diverse stages of the membrane fabrication and the cross-sectional view of the final composite membrane.

As can be seen, the external surface of the raw PSS support was formed by multiple stainless steel (SS) grains with diverse morphologies and sizes, thus generating a relatively high roughness and a wide pore size distribution of around a few microns, despite the limited original media grade of the supports (0.1 μm). These original large pore mouths were partially covered by mesoporous CeO_2_ particles, which formed the above-mentioned intermediate layer, shown as light grey areas with a noticeably amount of cerium and oxygen using EDS analysis (Table 1). This modified support presented a flatter surface with lower average pore sizes in comparison to the original one, which was more adequate for the subsequent deposition of palladium using ELP-PP. At this point, it should be remembered that mesoporous CeO_2_ particles were used to prepare this intermediate layer; therefore, porosity coming from the internal pores of these ceramic particles were added to the general macroporosity formed by the free spaces between the SS grains and the CeO_2_ particles. Moreover, the external surface of the final composite membrane after the incorporation of the Pd film using ELP-PP was also included. As expected, palladium was the main component (>90%), with other minor contributions of typical constituents of PSS and CeO_2_ and some carbon coming from the EDTA used during the plating step (Table 1).

An almost continuous external metal film could be distinguished despite feeding both a metal source and hydrazine as reducing agents from opposite sides of the porous substrate, and the chemical reaction preferentially took place inside the pores of the material. This has been widely explained for many other ELP-PP membranes in previous works by assuming a higher diffusion velocity of hydrazine toward the Pd source solution and a certain heterogeneity in the pores of the supports [8,17,28,30]. The smaller pores can be easily closed from the early stages of the ELP-PP process, while hydrazine can continue to pass through the larger pores, meeting the palladium ions on the external surface and generating the above-discussed external top film. It contains several cavernous pores that could be explained by the complete blockage of certain pores (the smallest ones) before completing the formation of the external film. This hypothesis can be supported by the cross-sectional views included in Figure 3, where a reasonably good homogeneity and continuity of the external film was found with a real thickness in the range 7–12 μm, which was very close to the one predicted using gravimetric analyses. This deviation was caused by the partial infiltration of Pd inside the pores of the support due to the nature of the ELP-PP method, which generates an irregular morphology on the interconnected surface between both Pd and PSS. This fact implies the generation of a Pd film with different surface morphologies at both the internal and external sides. The external surface was relatively flat and smooth, while the internal one, which was in contact with the porous substrate and materials of intermediate layers, had a noticeable tortuosity that increased the total available surface on this side. This fact was taken into account in the modeling to explain certain differences reached while permeating from the outer to the inner surface or vice versa. Further details about the morphology of this kind of membrane can be found elsewhere [29].

In addition to the morphological analysis, permeation tests are indispensable for completing the characterization of these membranes. A high ideal selectivity $\alpha_{H_{2}/N_{2}}\;\;$≥ 10,000 and a reference H_2_ permeance of around 1.03 × 10^−3^ mol·m^−2^·s^−1^·Pa^−0.5^ were reached at 375 °C. As usual for any other Pd composite membrane, this value varied with temperature according to an Arrhenius-type dependence with an activation energy of 8.91 kJ·mol^−1^ within the typical ranges. However, 17% higher H_2_ permeances were observed in the case of permeating from the inner to the outer surface of the membrane in contrast to the contrary operation, in which the permeate was collected from the lumen side. Moreover, a certain concentration polarization effect was also observed in the case of feeding H_2_/N_2_ mixtures instead of pure gases. In all cases, a slight deviation from the origin was observed when trying to fit the experimental data to the Sieverts’ law due to the above-mentioned partial infiltration of palladium into the pores of the substrate. A detailed explanation of the permeation behavior of this membrane can be found in our previous manuscript, where this ELP-PP membrane was presented for the first time [29]. Here, a brief summary of the most relevant parameters to be considered in the modeling is included. In this context, Table 2 displays the y-intercept and K values reached for the linear interpolation after considering each configuration and particular operating conditions through permeation experiments carried out at 375 °C (also detailed later in the CFD model). 

As it can be seen, the y-intercept values obtained for the permeation experiments carried out while permeating from the inner to the outer side of the membrane (in–out) were higher than those obtained for the contrary operation mode (out–in), as well as the *K* value in case of feeding pure hydrogen. However, an opposite trend was found for other *K* values obtained when feeding binary H_2_–N_2_ mixtures. This can be explained by the presence of the PSS support that affected the value measured for the partial hydrogen pressure only in the internal side of the Pd film and the total considered available membrane area on this side.

### 3.2. Porous Support Modeling

The porous model’s parameters that were established to characterize the PSS support were verified for two different pressure conditions and the in–out configuration. They were used for the rest of the simulations in which the Pd film for H_2_ separation was included. Table 3 displays the flow rates calculated with the model and those obtained during the experimental campaign carried out at 298 K. It should be highlighted that the calculated error was maintained below 1% in the case of operating at the highest pressure-driving force and, hence, permeate flow. However, this error increased up to 9% at lower pressures, with model predictions underestimating the real values reached from the experiments. This slight deviation was not significant, as the limiting step in this model was the H_2_ permeation through the Pd film, as governed by the Sieverts’ law.

**Figure 4:**
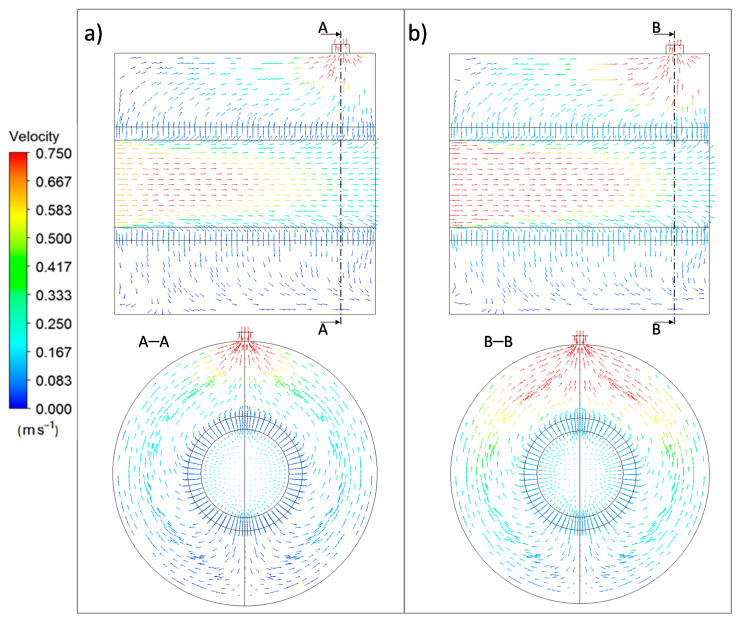
Velocity vectors for nitrogen permeation at 298 K through the PSS support (in–out configuration) at diverse pressure driving forces: (**a**) 0.5 bar and (**b**) 1.0 bar.

Figure 4 illustrates the behavior of the velocity vectors obtained for the permeation cell at the evaluated pressures. 

As can be observed, the velocity vectors traversed the porous support from the inlet section due to the adopted in–out configuration and surrounded the tubular shape to the outlet region in a similar manner independently of the considered pressure value. As expected, higher values of the velocity vectors could be observed for the highest pressure (1 bar). In any case, the support offered almost no resistance to the nitrogen pass throughout its structure, as expected by the relatively large pores of the porous stainless steel. This allowed a permeate flux of around 90% of the total feed while maintaining a pressure driving force between the retentate and permeate sides.

### 3.3. H_2_ Permeation through the Composite Membrane: In–Out Configuration

**Figure 5:**
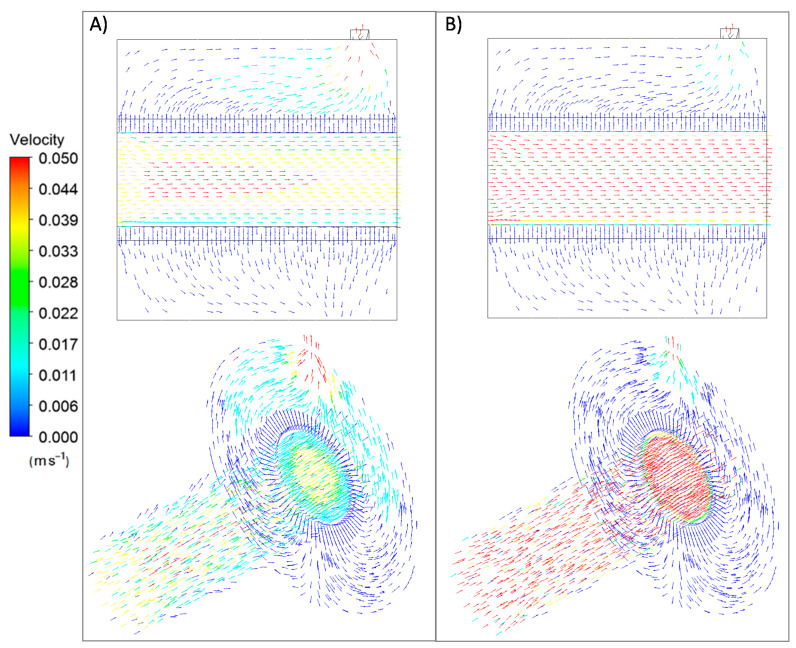
Modeled velocity vectors during permeation at *T* = 375 °C, Δ*P* = 2 bar, in–out configuration, and different H_2_ molar fractions in the feed stream: (**A**) 0.9 and (**B**) 0.5.

After dismissing the relevant additional resistance of the porous substrate to the gas permeation, the particular behavior reached after incorporating the H_2_-selective top Pd film was analyzed. In this context, Figure 5 shows the velocity vectors obtained during the permeation cases in which a constant pressure difference between the retentate and permeate sides was maintained at 2 bar, but two different binary H_2_–N_2_ mixtures were fed: one containing a high H_2_ molar fraction ($x_{H_{2}}\;$= 0.9, Figure 5a) and the other one at a moderate concentration level ($x_{H_{2}}\;$= 0.5, Figure 5b). 

In general, the velocity vectors crossed the composite membrane to the permeate side, decreasing the total permeate flux for lower H_2_ concentration levels. This fact can be explained by the intrinsic dilution effect under these conditions, consequently reducing the effective pressure driving force for the permeation process, which was calculated using the difference between the partial pressure of hydrogen at the retentate and permeate sides.

In contrast, the velocity values increased along the membrane’s axial direction more significantly when working with dilute fed mixtures. In these conditions, the lower permeate flux caused an increasing amount of non-permeated gases to reach the retentate outlet. However, this could not only be attributed to the dilution effect because the diverse N_2_ contents caused a variation of the H_2_ permeance despite considering the hydrogen partial pressures when calculating the pressure driving force used in Sieverts’ law. Therefore, some possible contribution of concentration polarization seems to be present, as previously suggested in [29]. In any case, this behavior is coherent with the H_2_ permeance values reached under both conditions, with them being around 2.5 times higher in the case of feeding H_2_ concentrate mixtures instead of the diluted one.

**Figure 6:**
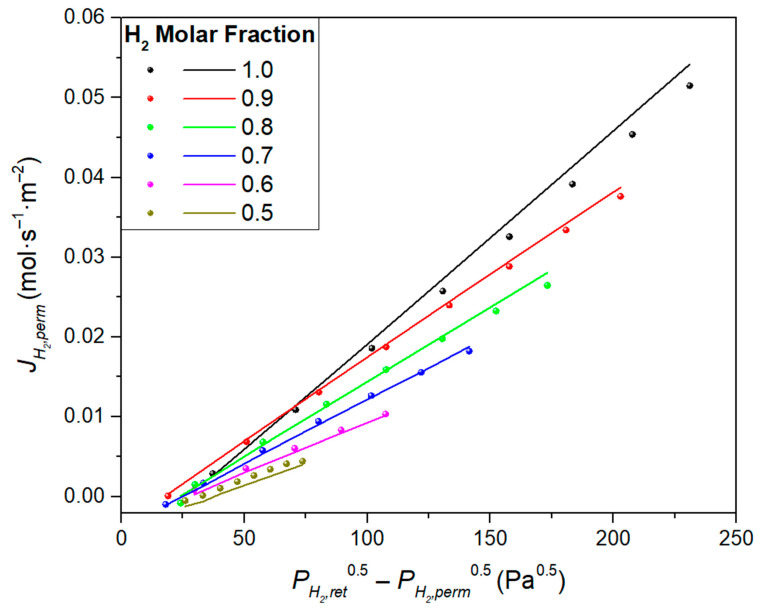
Experimental (symbols) and predicted (lines) H_2_ permeate fluxes at a constant temperature (*T* = 375 °C) but different pressures and H_2_ molar fractions in the feed stream (in–out configuration).

The H_2_ permeate flux reached for the entire range of operating conditions for the in–out configuration is shown in Figure 6, which compares the simulated (lines) and experimental (circles) data.

Slight deviations at feed molar fractions of 1 and 0.5 can be observed although, in general, the agreement between experimental and modeled data was quite good with simulated permeance deviations were below 6%. This could be attributed to the fact that experimental H_2_ permeance values were included in the CFD model; therefore, it was not used strictly as a predictive tool. However, it is worth noticing that the CFD considered the local gradients of pressure and concentration for each cell, and Sieverts’ law was locally computed for the closest regions to the Pd film. In contrast, for the experimental fitting, an overall H_2_ partial pressure was used for each particular case. Based on this fact, more significant differences could be expected for broad partial pressure ranges. In this case, the model led to accurate results when computing local gradients due to the porous support being correctly modeled, and precise local pressures only on the membrane side can be predicted.

**Figure 7:**
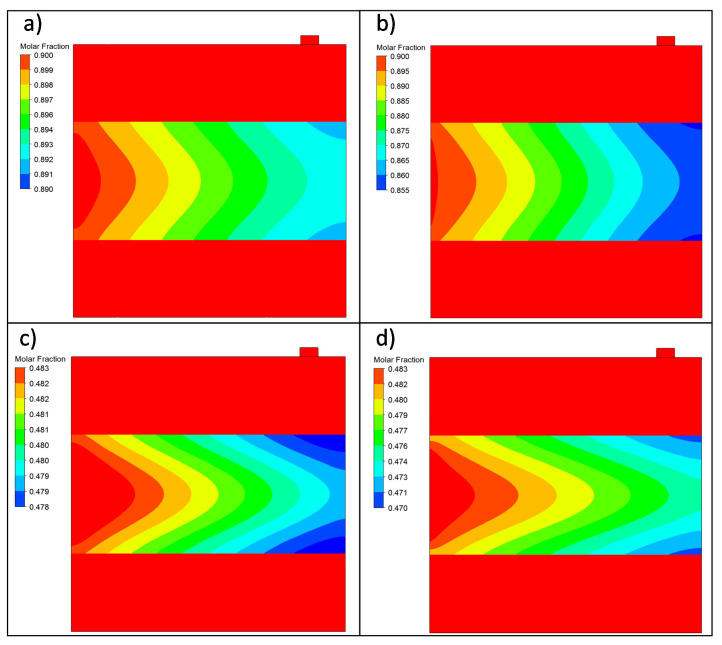
H_2_ molar fraction variation in the symmetry plane for the in–out permeation experiments at a constant temperature (*T* = 375 °C) but various feeding conditions: (**a**) $x_{H_{2}}^{feed}\;$= 0.9 and Δ*P* = 0.5 bar, (**b**) $x_{H_{2}}^{feed\;}$= 0.9 and Δ*P* = 2 bar, (**c**) $x_{H_{2}}^{feed}\;$= 0.5 and Δ*P* = 1.4 bar, and (**d**) $x_{H_{2}}^{feed\;}$= 0.5 and Δ*P* = 2 bar.

After addressing the effect of working with gas mixtures with diverse concentrations, Figure 7 contains the results reached when analyzing the composite ELP-PP membrane’s permeation behavior when the H_2_ feed molar fraction was maintained at a constant value but the pressure driving force varied. In this manner, the results obtained while operating at 0.5 and 2.0 bar when feeding a binary gas mixture with constant $x_{H_{2}}$ = 0.9 are shown in Figure 7a,b, respectively. A similar study was performed in the case of feeding diluted mixtures with $x_{H_{2}}$ = 0.5 and varying the pressure between 1.4 and 2.0 bar (Figure 7c,d, respectively). 

First, it should be noted that, based on previous experimental results, an eventually complete selectivity of the Pd film was considered. Therefore, a constant $x_{H_{2}}$ = 1.0 was maintained for the entire permeate side independently of the modeled experimental conditions. At this point, it can be observed that the concentration profiles inside the retentate side (the differences in concentration along the inner diameter) were more pronounced for a feed concentration of 0.5, independently of the applied transmembrane pressure. The presence of N_2_ in the feed steam increased the polarization effect, resulting in a reduction in the driving force and, consequently, in the permeated hydrogen flow.

### 3.4. H_2_ Permeation through the Composite Membrane: Out–In Configuration

**Figure 8:**
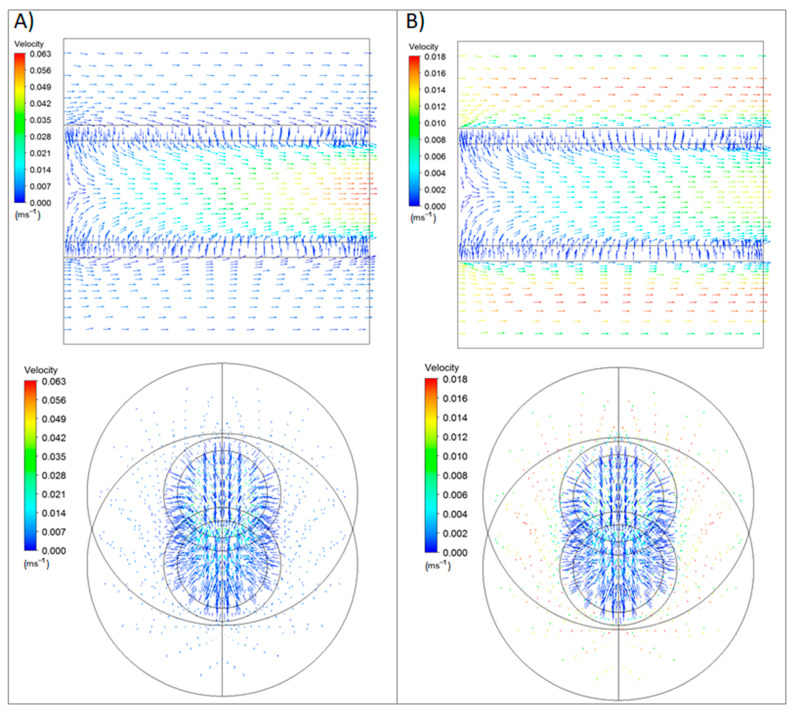
Velocity vectors for the out–in configuration at a constant temperature (*T* = 375 °C) and feed pressure (Δ*P* = 2 bar), but two different H_2_ molar fraction: (**A**) 0.9 and (**B**) 0.5.

In this section, the effect of permeating according to the contrary direction of the permeate flux is addressed. First, the behavior of the velocity vectors was evaluated for the new configuration in Figure 8.

In both cases, the permeate flow was higher for the out–in configuration, as hydrogen on the retentate side did not have the porous steel’s intermediate resistance before reaching the palladium surface. This particular effect, not observed in the case of operating in the contrary mode, was due to the presence of the porous substrate and a more tortuous Pd surface on the permeate side reduced the concentration polarization effect on the feed side where the hydrogen and nitrogen coexisted. In this case, both gases met first at the outer and smoother surface of the palladium, where the non-permeated nitrogen was removed in an easier manner from the metal surface.

**Figure 9:**
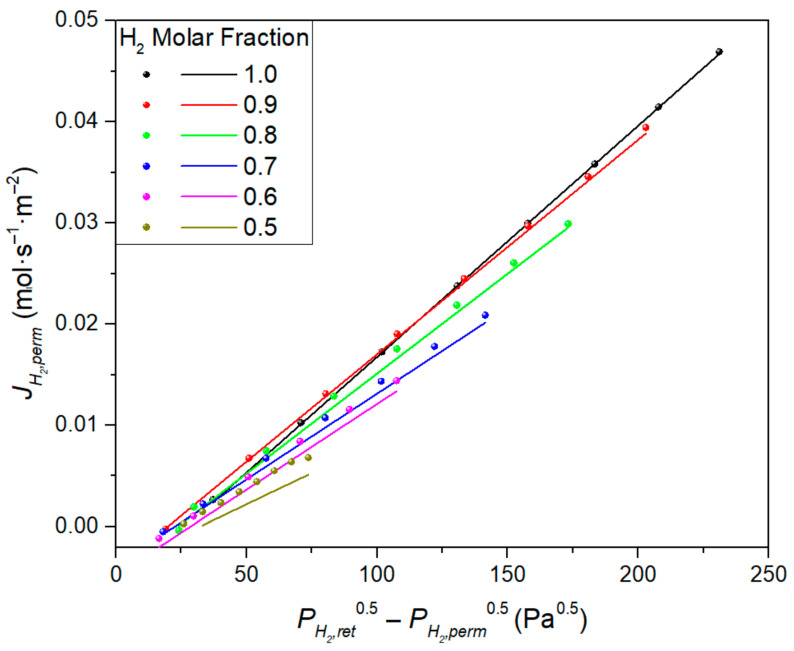
Experimental (symbols) and predicted (lines) H_2_ permeate fluxes at a constant temperature (*T* = 375 °C) but different pressures and H_2_ molar fractions in the feed stream (out–in configuration).

The H_2_ permeate flux simulation results were compared with the experimental ones for the out–in configuration in Figure 9. The simulations tended to underestimate the H_2_ permeate flux at low molar fraction feeds. A better agreement at high molar fractions was reached. These simulation series achieved excellent calculated permeances that were in agreement compared with the experiments, with deviations below 2%. Only the 0.5 molar fraction series had a worse agreement, with a permeance deviation of 13%.

**Figure 10:**
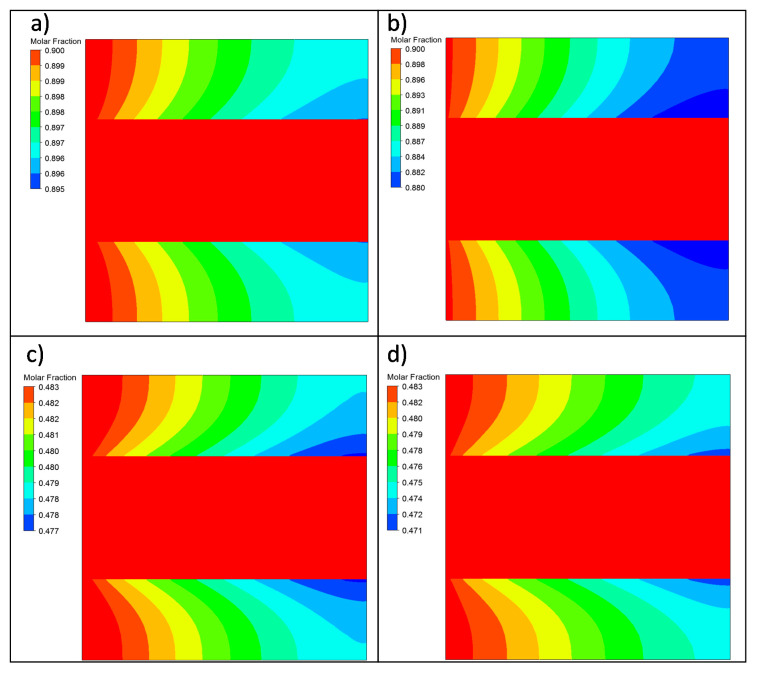
H_2_ molar fraction variation in the symmetry plane for the out–in permeation experiments at a constant temperature (*T* = 375 °C) but with various feeding conditions: (**a**) $x_{H_{2}}^{feed\;}$= 0.9 and Δ*P* = 0.5 bar, (**b**) $x_{H_{2}}^{feed}\;$= 0.9 and Δ*P* = 2 bar, (**c**) $x_{H_{2}}^{feed}\;$= 0.5 and Δ*P* = 1.4 bar, and (**d**) $x_{H_{2}}^{feed}\;$= 0.5 and Δ*P* = 2 bar.

Figure 10a,b show, respectively, the results while operating at 0.5 and 2.0 bar when feeding a binary gas mixture with a constant $x_{H_{2}}$ = 0.9. In the same way, the results obtained when the system was fed with a constant $x_{H_{2}}$ = 0.5 at 1.4 and 2.0 bar are presented in Figure 10c,d, respectively.

The H_2_ permeation simulations showed interesting differences between the in–out and out–in configurations. In the H_2_ molar fraction contour, both systems presented the concentration polarization phenomenon, where the H_2_ concentration gradient near the membrane obstructed the hydrogen permeation. In all contour plots, two different gradients were observed: one in the axial direction due to the disappearance of H_2_ from the retentate region and the other one in the x-direction near the membrane’s surface as N_2_ occupied the volume freed by H_2_, hindering the H_2_ transport to the membrane and decreasing the permeation capacity.

The concentration polarization in the in–out configuration was more significant than in the out–in one, as shown in the contour plots. The H_2_–N_2_ mixture needed to pass through the porous steel first to reach the membrane and, when the H_2_ permeated, the N_2_ could not return to the inner region. This phenomenon was less apparent in the out–in configuration because the outer region was directly connected with the membrane.

### 3.5. General Model Validation

**Figure 11:**
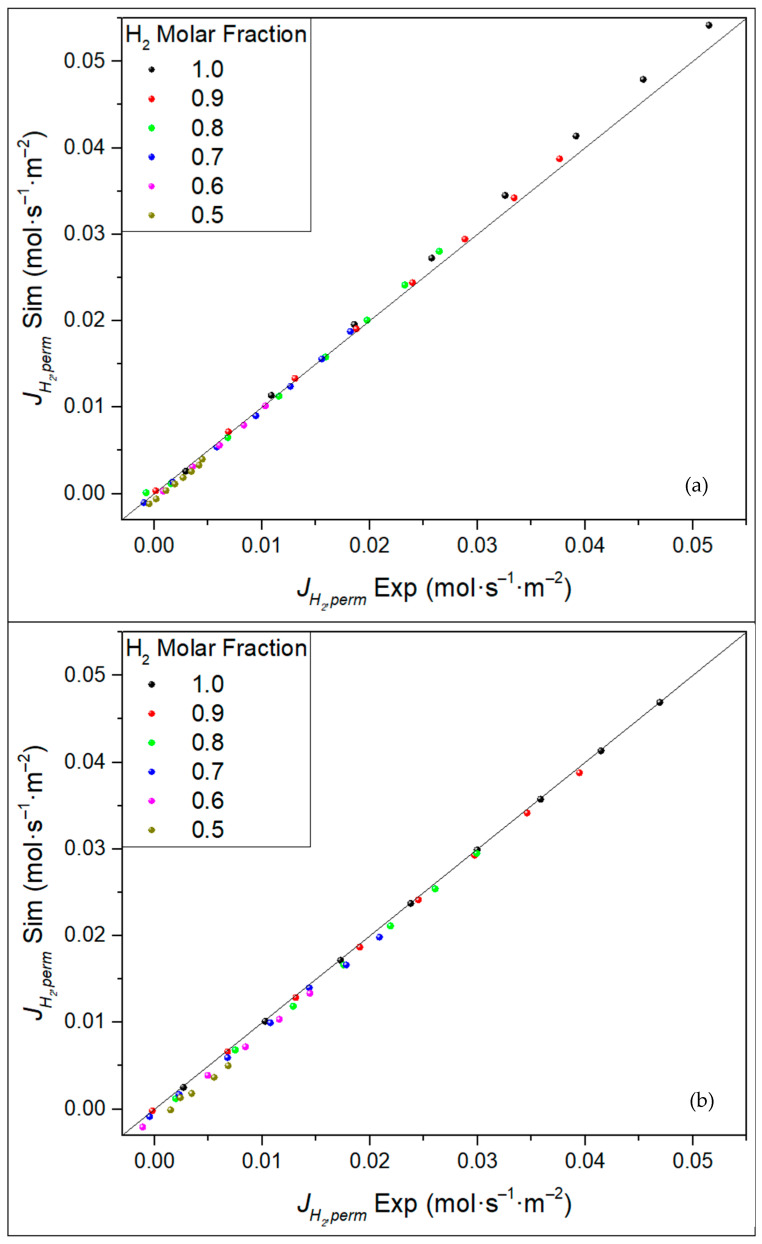
Parity plot for simulated permeation results against experimental ones for all the considered H_2_ feed molar fractions for different operating modes: (**a**) in–out and (**b**) out–in.

Finally, a general model validation is presented in Figure 11, collecting both the experimental and modeled permeate fluxes for all the analyzed conditions as a parity plot. In general, an average error below 4% was maintained between both the experimental and modeled permeances, with the highest permeance error getting close to 13% only in the lowest molar fraction series while considering the out–in configuration. However, the plot evidenced a trend of underestimating the H_2_ permeation in the lower feed molar fractions range. Some explanations can be provided to explain this trend. Effects like pressure drops along the retentate side or local variations in the H_2_ molar fraction are not considered in experimental calculations. Even though pressure drops in the simulations are almost negligible, below 4%, some that are large enough to influence the results are still possible when compared with experiments. Finally, local variations in the H_2_ molar fraction could also be a source of little deviations between the simulations and experiments.

## 4. Conclusions

The porous model based on the Darcy–Forcheimer formulation seemed to be a good approximation to describe the flow through the PSS supports. The experimental data of the permeate fluxes successfully validated the model’s predictions for different flow rates and nitrogen feed pressures. The combination of the porous model and Sieverts’ law using a source–sink pair approach showed good accuracy in the simulations of H_2_ permeation for supported ELP-PP palladium-based membranes for a wide range of conditions. Predictions for the H_2_ permeation fluxes showed a higher effect of concentration polarization in the in–out configuration due to the greater difficulties the new hydrogen had reaching the Pd film and the non-permeated nitrogen had returning toward the retentate bulk phase. In contrast, this process was much easier while operating in the out–in mode due to the smoother external surface of the Pd film. In this case, the tortuosity of the porous substrate had to be passed through by pure hydrogen, which did not affect the transport of the gas from the feed stream, in which a gas mixture was present, to the Pd film.

By considering a simulated feed that reproduced the conditions of experimental data of a specific ELP-PP membrane, the model allowed for predicting the molar fraction profiles along the reactor, and hence, the performance of the membrane as a separation device. These results are very useful for calculating the optimal length and mechanical design of the permeator, where the concentration polarization near the membrane surface is not a limit for the hydrogen permeation. However, a more detailed mechanistic description of the hydrogen permeation is required to understand the influence of each fundamental step, which even involves considering the presence of inhibiting species, such as carbon monoxide or steam. Future efforts will be focused on implementing a more rigorous permeation model for these particular ELP-PP membranes, in which both sides of the palladium film are noticeably different. In the meantime, this first simplified model can reproduce the H_2_ permeation through ELP-PP composite Pd-based membranes formed by diverse stacked layers with reasonable accuracy, evidencing how these numerical models are an excellent opportunity to start new studies about these membranes’ permeation behavior under diverse operating conditions.

## Figures and Tables

**Table 1:** Membrane elemental composition that was found using EDS at diverse stages of the fabrication process.

Sample	Elemental Composition by EDS (wt.%)								
	Fe	Cr	Ni	Mo	C	O	Ce	Pd	Others
Raw PSS	63.5	17.0	9.0	2.0	<0.03	-	-	-	8.5
Calcined PSS	60.1	8.8	4.6	1.5	1.3	23.3	-	-	0.4
CeO_2_/PSS	48.08	6.2	2.4	0.5	1.8	28.94	11.8	-	0.3
Pd/CeO_2_/PSS	0.7	-	-	-	5.3	0.3	0.9	92.8	-

**Table 2:** Main permeation parameters at 375 °C and different H_2_ feed molar fractions (*X_H2_*) obtained from experimental data: y-intercept and H_2_ permeance (*K*).

***X_H2_* (Feed)**	**y-Intercept × 10^6^** **(kg·m^−2^·s^−1^)**	***K* × 10^7^** **(kg·m^−2^·s^−1^·Pa^−0.5^)**
0.5	−6.601	2.179
0.6	−5.380	2.446
0.7	−6.784	3.102
0.8	−7.962	3.597
0.9	−6.965	4.085
1.0	−13.680	5.023
***X_H2_*** **(Feed)**	**y-Intercept × 10^6^** **(kg·m^−2^·s^−1^)**	***K* × 10^7^** **(kg·m^−2^·s^−1^·Pa^−0.5^)**
0.5	−6.505	2.827
0.6	−7.843	3.450
0.7	−6.846	3.476
0.8	−8.547	3.996
0.9	−8.461	4.306
1.0	−11.848	4.562

**Table 3:** Comparison of the experimental and predicted values of permeate flow for pure N_2_ streams for different operating conditions with the PSS support.

ΔP (bar)	Feed (g/s)	Permeation (g/s)		Error (%)
		Experimental	Model	
0.5	0.0195	0.0173	0.0158	− 8.58
1	0.0400	0.0373	0.0376	0.96

## Data Availability

Supporting dataset available at https://doi.org/10.5281/zenodo.4516231.

## Supplementary Materials

The following are available online at https://www.mdpi.com/2077-0375/11/2/123/s1.
