# Supplementary material for: Modeling of H2 Permeation through Electroless Pore-Plated Composite Pd Membranes Using Computational Fluid Dynamics
Source: Membranes (Basel). 2021 Feb 9;11(2):123. doi: 10.3390/membranes11020123 (PMC7915706; doi:10.3390/membranes11020123)
Supplement: Supplementary file 1 [file membranes-11-00123-s001.pdf]

membranes

Supplementary Information: Modeling of H_2-Permeation Through Electroless Pore-Plated Composite Pd-Membranes by Computational Fluid-Dynamics

Alberto Fernández, Cintia Casado, David Alique, José Antonio Calles and Javier Marugán

User-defined functions (UDF) used in our model are presented below. The first one, called "Adjacent Membrane's Cell Finder UDF", shows how to find, for the studied geometry, adjacent cells to palladium membrane and how to get its area and volume. Area values are stored as negative values with a minus sign for the retentate side. Also, the ID of each cell is stored to be used in the next UDF.

The second one, called "Source-Sink Pair UDF", calculates, for each adjacent cell to the palladium membrane, the sink and source values of hydrogen flow using the Sieverts Law adapted to this model (Equation (6) in the article). This calculation iterates using the molar fractions and the hydrogen pressures at both sides along the system. Also, it uses the experimental membrane's permeance and Y-intercept values. The values of each cell's area and volume are taken from the previous UDF, as they were stored in user-defined memories (UDM's) accessible from other UDF's.

The third UDF is used to calculate the hydrogen diffusion coefficient and, the last one, to calculate the viscosity of the mixture.

```
//ADJACENT MEMBRANE'S CELLS FINDER UDF
//UDMI(0) stores the area of each cell, negative area in the Sink side, positive in the Source side
//UDMI(1) stores the ID of each cell opposite to each cell near the membrane surface
#include "udf.h"
DEFINE_ON_DEMAND(Cell_Finder)
{
Domain *d; /* Get the domain using Fluent utility */
face_t f;
cell_t c, c0;
real c1;
real NV_VEC(fArea);

Thread *tPD, *tPD_s, *tOutside, *tInside, *ct;

//ID for palladium and palladium shadow boundaries from Fluent.
int Pd_ID = 10;
int PdShadow_ID = 34;
d = Get_Domain(1);

/* Initialise Cells */
/* this loops over all cells and lets the UDM = 0 */
thread_loop_c(ct, d)
{
begin_c_loop(c,ct)
```

```
          {
      C_UDMI(c,ct,0) = 0.0;
          }
      end_c_loop(c,ct)
      }

//Store the domains to each side from the membrane interface
tPD_s = Lookup_Thread(d, PdShadow_ID);
tInside = THREAD_T0(tPD_s);
tPD = Lookup_Thread(d, Pd_ID);
tOutside = THREAD_T0(tPD);

//this loops over the outer side
begin_f_loop(f,tPD)
      {
//c0 is the adjacent cell to the interface on the outer side
//c1 is the adjacent cell on the inner side
c0 = F_C0(f, tPD);
c1 = F_C0(f, tPD_s);
F_AREA(fArea,f,tPD);
/* this loops over all cells adjacent to wall and lets the UDM = Cell_Area */
C_UDMI(c0, tOutside, 0) = NV_MAG(fArea);
C_UDMI(c0,tOutside,1) = c1;
      }
end_f_loop(f,tPD)

//this loops over the internal side
begin_f_loop(f,tPD_s)
      {
//c0 is the adjacent cell to the interface on the inner side
//c1 is the adjacent cell on the outer side
c0 = F_C0(f, tPD_s);
c1 = F_C0(f, tPD);
F_AREA(fArea,f,tPD_s);
/* this loops over all cells adjacent to wall and lets the UDM = -Cell_Area */
C_UDMI(c0, tInside, 0) = -NV_MAG(fArea);
C_UDMI(c0,tInside,1) = c1;
      }
end_f_loop(f,tPD_s)
}

//SOURCE-SINK PAIR UDF
//UDMI(0) stores the area of each cell, negative area in the Sink side, positive in the Source side
```

```
//UDMI(1) stores the ID of each cell opposite to each cell near the membrane surface
//UDMI(2) stores SOURCE-SINK value
#include "udf.h"
#define KSIEVERT 2.1792E-07 //Permeance value for each molar fraction experiments, kg·m^-2·s^-1·Pa^-0.5
#define YVALUE 6.6014E-06 //Y-intercept value for each molar fraction experiments, kg·m^-2·s^-1
DEFINE_SOURCE(h2_sink, c, t, dS, eqn)
{
Domain *d; /* Get the domain using Fluent utility */
cell_t  c0;
Thread  *tOutside;
real source;
real mole_fraction1;
d = Get_Domain(1);
//Get the external domain. t it´s from inside
tOutside = Lookup_Thread(d,8);
source=0.0;
dS[eqn]=0;
mole_fraction1=C_YI(c,t,0)/2.0/(C_YI(c,t,0)/2.0+(1.0-C_YI(c,t,0))/28.0);
if (C_UDMI(c,t,0) <0.0)
     {
if(C_P(c,t)+101325.0>0.0 && (C_P((int)(C_UDMI(c,t,1)),tOutside)+101325.0)>0.0)
                {
source=C_UDMI(c,t,0)*KSIEVERT*(sqrt(mole_fraction1*(C_P(c,t)+101325.0))-sqrt((C_P((int)(C_UDMI(c,t,1)),tOutside)+101325.0)))/C_VOLUME(c,t)
-C_UDMI(c,t,0)/C_VOLUME(c,t)*YVALUE;
if((eqn) && C_YI(c,t,0)>0.0)         dS[eqn]=C_UDMI(c,t,0)*KSIEVERT*0.5*(C_P(c,t)+101325.0)/(sqrt(mole_fraction1*(C_P(c,t)+101325.0))*C_VOLUME(c,t));
                }
     }
C_UDMI(c,t,2)=source;
return source;
}
DEFINE_SOURCE(h2_source, c, t, dS, eqn)
{
Domain *d; /* Get the domain using Fluent utility */
//cell_t  c0;
Thread  *tInside;
real source =0.0;
real mole_fraction2;
dS[eqn]=0;
//int Pd_ID = 3;
//int PdShadow_ID = 7;
d = Get_Domain(1);
```

```
//Get the external domain. t it´s from outside
tInside = Lookup_Thread(d,7);
mole_fraction2=C_YI((int)(C_UDMI(c,t,1)),tInside,0)/2.0/(C_YI((int)(C_UDMI(c,t,1)),tInside,0)/2.0+(1.0-
C_YI((int)(C_UDMI(c,t,1)),tInside,0))/28.0);
if (C_UDMI(c,t,0) >0.0)
      {
if(C_P(c,t)+101325.0>0.0 && (C_P((int)(C_UDMI(c,t,1)),tInside)+101325.0)>0.0)
source=C_UDMI(c,t,0)*KSIEVERT*(sqrt(mole_fraction2*
(C_P((int)(C_UDMI(c,t,1)),tInside)+101325.0))-sqrt((C_P(c,t)+101325.0)))/C_VOLUME(c,t)-C_UDMI(c,t,0)/C_VOL-
UME(c,t)*YVALUE;
if((eqn) && C_YI((int)(C_UDMI(c,t,1)),tInside,0)>0.0)
dS[eqn]=C_UDMI(c,t,0)*KSIEVERT*0.5*(C_P((int)(C_UDMI(c,t,1)),tInside)+101325.0)/(sqrt(mole_fraction2*
(C_P((int)(C_UDMI(c,t,1)),tInside)+101325.0))*C_VOLUME(c,t));
                 }
      }
C_UDMI(c,t,2)=source;
return source;
}

//HYDROGEN DIFFUSIVITY UDF
#include "udf.h"

//DEFINE_DIFFUSIVITY(diff,c,t,i)
{
real diff; /*Hydrogen diffusivity in m^2/s*/
diff=1.236E-25*pow(C_P(c,t)+101325.0,4.0)-1.222E-19*pow(C_P(c,t)+101325.0,3.0)+4.673E-
14*pow(C_P(c,t)+101325.0,2.0)-8.613E-09*(C_P(c,t)+101325.0)+7.631E-04;
return diff;
}
//MIXTURE VISCOSITY UDF
#include "udf.h"
DEFINE_PROPERTY(cell_viscosity, c, t)
{
real mu; /*mixture viscosity in Pa·s*/
real w; /*Hydrogen molar fraction*/
w = C_YI(c,t,0)/2.0/(C_YI(c,t,0)/2.0+(1.0-C_YI(c,t,0))/28.0);
           mu= -2.708E-05*pow(w,4.0)+2.902E-05*pow(w,3.0)-1.741E-05*pow(w,2.0)-3.113E-07*w+3.098E-05;
return mu;
}
```
